# Identification and Verification of the Prodigiosin Biosynthetic Gene Cluster (BGC) in Pseudoalteromonas rubra S4059

**DOI:** 10.1128/Spectrum.01171-21

**Published:** 2021-09-29

**Authors:** Xiyan Wang, Thomas Isbrandt, Emil Ørsted Christensen, Jette Melchiorsen, Thomas Ostenfeld Larsen, Sheng-Da Zhang, Lone Gram

**Affiliations:** a Department of Biotechnology and Biomedicine, Technical University of Denmark, Lyngby, Denmark; University of Minnesota

**Keywords:** biosynthetic gene cluster, di-pyrrolyl-dipyrromethene prodigiosin, prodigiosin, *Pseudoalteromonas rubra*

## Abstract

Pseudoalteromonas rubra S4059 produces the red pigment prodigiosin, which has pharmaceutical and industrial potential. Here, we targeted a putative prodigiosin-synthesizing transferase PigC, and a *pigC* in-frame deletion mutant did not produce prodigiosin. However, extractions of the *pigC* mutant cultures retained antibacterial activity, and bioassay-guided fractionation found antibacterial activity in two fractions of blue color. A precursor of prodigiosin, 4-methoxy-2,2′-bipyrrole-5-carbaldehyde (MBC), was the dominant compound in both the fractions and likely caused the antibacterial activity. Also, a stable blue pigment, di-pyrrolyl-dipyrromethene prodigiosin, was identified from the two fractions. We also discovered antibacterial activity in the sterile filtered (nonextracted) culture supernatant of both wild type and mutant, and both contained a heat-sensitive compound between 30 and 100 kDa. Deletion of prodigiosin production did not affect growth rate or biofilm formation of P. rubra and did not change its fitness, as the mutant and wild type coexisted in equal levels in mixed cultures. In conclusion, a prodigiosin biosynthetic gene cluster (BGC) was identified and verified genetically and chemically in *P. rubra* S4059 and a stable blue pigment was isolated from the *pigC* mutant of S4059, suggesting that this strain may produce several prodigiosin-derived compounds of pharmaceutical and/or industrial potential.

**IMPORTANCE** Pigmented *Pseudoalteromonas* strains are renowned for their production of secondary metabolites, and genome mining has revealed a high number of biosynthetic gene clusters (BGCs) for which the chemistry is unknown. Identification of those BGCs is a prerequisite for linking products to gene clusters and for further exploitation through heterologous expression. In this study, we identified the BGCs for the red, bioactive pigment prodigiosin using genomic, genetic, and metabolomic approaches. We also report here for the first time the production of a stable blue pigment, di-pyrrolyl-dipyrromethene prodigiosin (Dip-PDG), being produced by the *pigC* mutant of Pseudoalteromonas rubra S4059.

## INTRODUCTION

Many secondary metabolites from microorganisms and plants have bioactivities of pharmaceutical and agricultural interest. For instance, more than 60% of the antibiotics used in the clinic have been derived from microorganisms, notably soil-dwelling actinobacteria (1). The current antibiotic crisis is driven both by the rapid development and spread of antibiotic resistance in pathogenic bacteria and by the lack of discovery of novel antibiotic compounds (2). We are thus in urgent need of discovering novel antibiotics, and exploring microorganisms from new environments is one of the promising strategies to uncover novel antibiotics. Although many bioactive compounds have been discovered from marine (micro)organisms, such as antibiotics from the marine genus *Pseudoalteromonas*, the marine environment remains a less-explored drug discovery reservoir compared to soil.

*Pseudoalteromonas* species are strictly marine bacteria and can be divided into two groups, pigmented and nonpigmented strains (3), and especially the pigmented strains can produce many bioactive compounds (3, 4). Recently, genome mining of several *Pseudoalteromonas* species indicated that the genetic potential for secondary metabolite production of several pigmented species is equal to that of the *Actinobacteria*. For instance, a red-pigmented Pseudoalteromonas rubra strain S4059 dedicates as much as 15% of its genome to gene products likely involved in secondary metabolite production and contains 19 biosynthetic gene clusters (BGCs) (5). However, only one compound class, namely, prodiginines, has been chemically identified in this strain, and the BGC responsible for the production of the red pigment in S4059 is not known (4).

Prodigiosin is a member of the prodiginine family, which is characterized by a tri-pyrrole structure. Prodigiosin consists of two moieties, 2-methyl-3-*n*-amyl-pyrrole (MAP) and 4-methoxy-2,2′-bipyrrole-5-carbaldehyde (MBC), which are linked via a condensation reaction to form the final product. Several prodigiosin analogues have been described, varying mostly in the length of the aliphatic side chain on the MAP moiety (6). The two moieties are coupled by a synthesizing transferase PigC in a final condensation step to form prodigiosin (7). Prodigiosin was first discovered from a terrestrial bacterium Serratia marcescens (8), has since been found in several other bacterial species, such as Hahella chejuensis (6), Streptomyces coelicolor (9), and Pseudoalteromonas rubra (4, 10), and has attracted attention due to its multiple activities, having antibacterial (11), anticancer (12), algicidal (13), and immunosuppressive activity (14). The prodigiosin derivatives are also of interest; for instance, cycloprodigiosin, which was isolated from *P*. *rubra* and is more active against Staphylococcus aureus than prodigiosin (15). Interestingly, most of the prodiginines are red pigments; however, di-pyrrolyl-dipyrromethene prodigiosin (Dip-PDG), which has been reported in only few bacteria species, such as Hahella chejuensis (6) and a mutant S. marcescens strain (16), is a blue pigment, due to having a longer conjugated chromophore system.

Although prodigiosin exhibits cytotoxicity against eukaryotic cells (chick embryos) (8) and thus is not deemed suitable as a clinical drug, prodigiosin has recently received renewed attention because prodigiosin and its derivatives are effective proapoptotic agents against cancer cell lines with no or little toxicity against normal cell lines (8). This finding indicates that prodigiosin could be a promising drug candidate and further identification of the BGC is the prerequisite to heterologous expression that has allowed production of compounds from difficult-to-culture strains. Also, given the large number of BGCs and potential for production of other compounds, genetic manipulation of the organism is required to determine the link between particular BGCs and metabolomic profile. We therefore selected a pigmented P. rubra S4059, which can produce prodigiosin, to identify the prodigiosin BGC and query if this strain could produce other bioactive compounds.

## RESULTS

### A prodigiosin BGC was identified and verified in Pseudoalteromonas rubra S4059.

**FIG 1:**
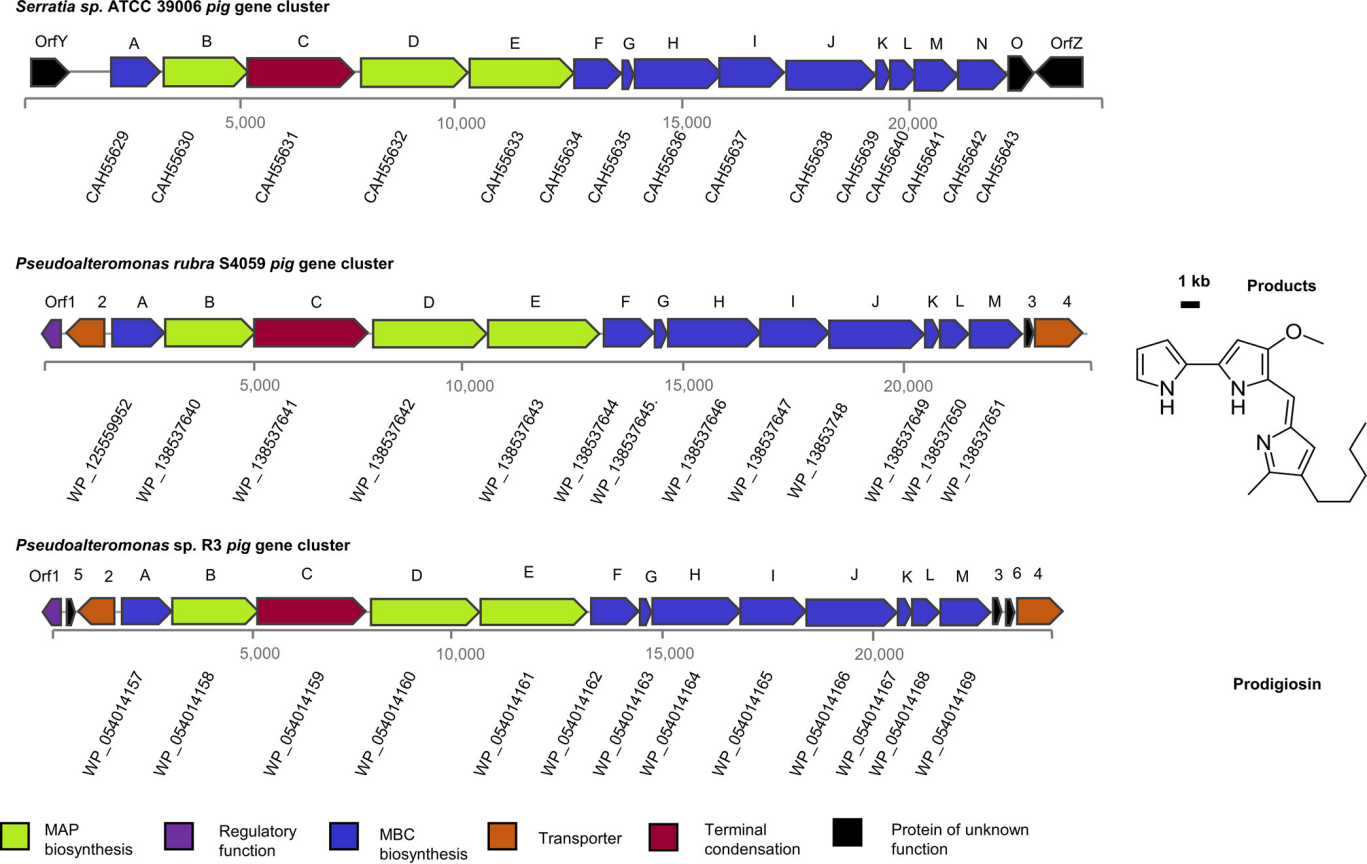
The genetic organization of prodigiosin biosynthetic gene clusters of *Serratia* sp. ATCC 39006, Pseudoalteromonas rubra S4059, and *Pseudoalteromonas* sp. R3. Block arrows are color coded to show which genes encode proteins involved in the biosynthesis of prodigiosin, and the accession numbers of the genes in each species were listed under each single gene.

Vynne et al. have demonstrated that *P. rubra* S4059 can produce the red pigment prodigiosin (4); however, the BGC responsible for prodigiosin production in S4059 has not been identified. A putative prodigiosin-synthesizing transferase PigC was annotated in S4059 by Prokka (17), and its surroundings genes are putative prodigiosin biosynthesis-associated genes. Further, antiSMASH 6.0 (1V) was used to tentatively predict the prodigiosin BGC in S4059 and resulted in the same BGC as that annotated by Prokka. To further characterize the prodigiosin BGC in S4059, the putative prodigiosin-associated genes in S4059 were compared to the known prodigiosin BGC in *Serratia* sp. ATCC 39006 and that in *Pseudoalteromonas* sp. R3 (Fig. 1; Table S1). The prodigiosin BGC in ATCC 39006 contains 14 genes, *pigA* to *pigO* (Fig. 1) (18), while the putative prodigiosin BGC in S4059 and R3 consists of 13 genes that are arranged *pigA* through to *pigM* (Fig. 1). The *Pseudoalteromonas pig* genes are similar to the genes in the ATCC 39006 *pig* gene cluster, except that no homologs of *pigN* and *pigO* are apparently present (Fig. 1). The relatedness between each of the 13 prodigiosin proteins found in S4059 and their homologs in the prodigiosin BGC of R3 and ATCC 39006 was compared (Table S1). Proteins encoded by the genes in the prodigiosin BGC show a wide variation in the level of identity. For example, the ATCC 39006 PigE homologs in S4059 were on average 79.3% identical, while the PigL homologs on average were only 37.63% identical (Table S1).

**FIG 2:**
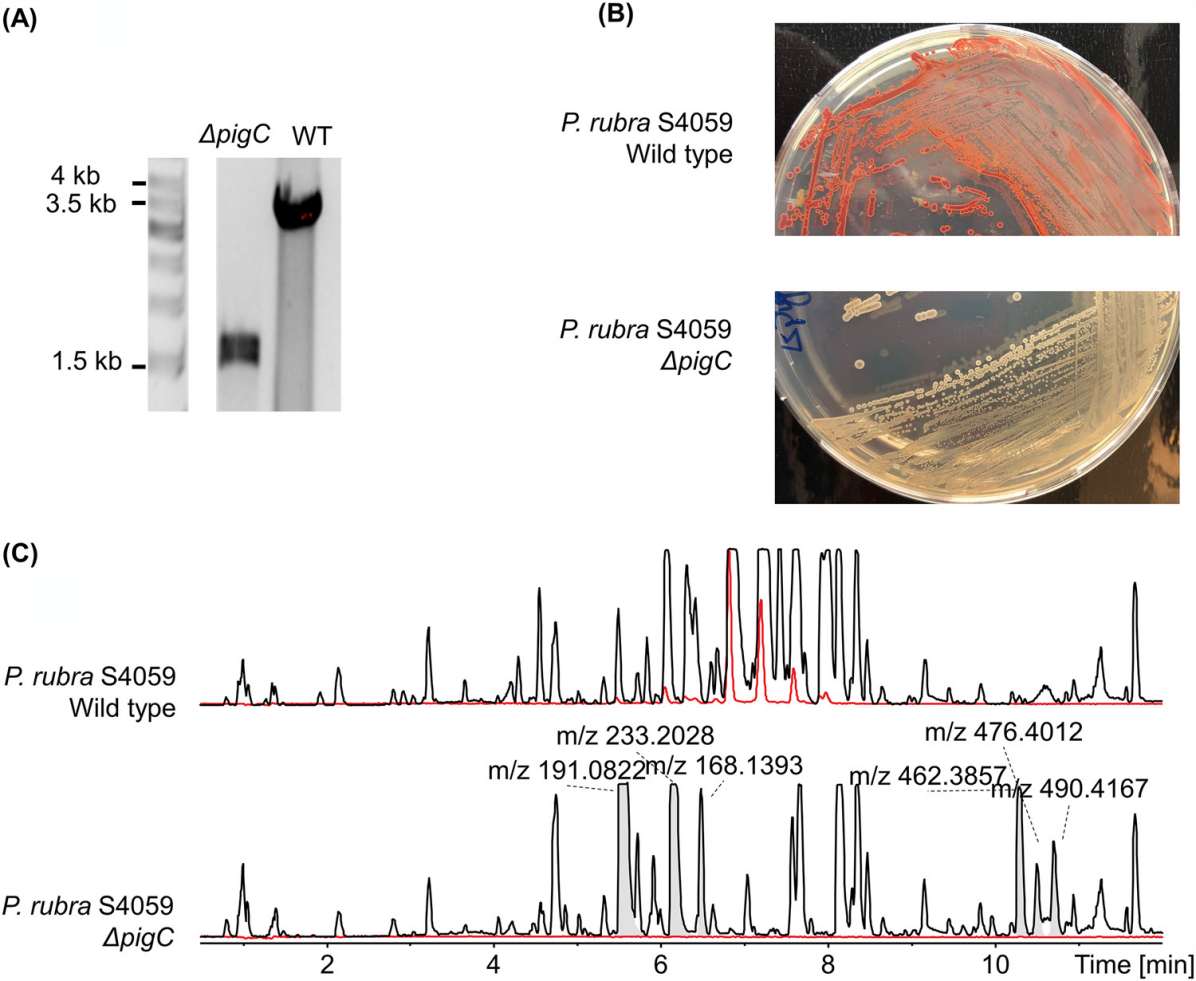
The *pigC* gene encodes a prodigiosin-synthesizing transferase. A prodigiosin-deficient mutant was generated by knocking out *pigC* gene (A), and the phenotype of Δ*pigC* is brownish (B). The LC-MS profiles (C) of *P. rubra* S4059 and prodigiosin-deficient mutant Δ*pigC.* The red peaks are prodigiosin and its analogs.

To verify the prodigiosin BGC in S4059, the putative prodigiosin-synthesizing transferase *pigC* was selected as a target gene to generate an in-frame deletion mutant (Fig. 2A). Colonies of Δ*pigC* were slightly brownish but did not produce a red pigment (Fig. 2B). The chemical profile of Δ*pigC* was analyzed by liquid chromatography-mass spectrometry (LC-MS) and prodigiosin was not produced in the Δ*pigC* mutant (Fig. 2C). In conclusion, the prodigiosin-related BGC was identified and verified in *P. rubra* S4059 by genomic, genetic, and metabolomic evidence.

### Deletion of a *pigC* gene does not influence the growth and biofilm formation ability of S4059.

**FIG 3:**
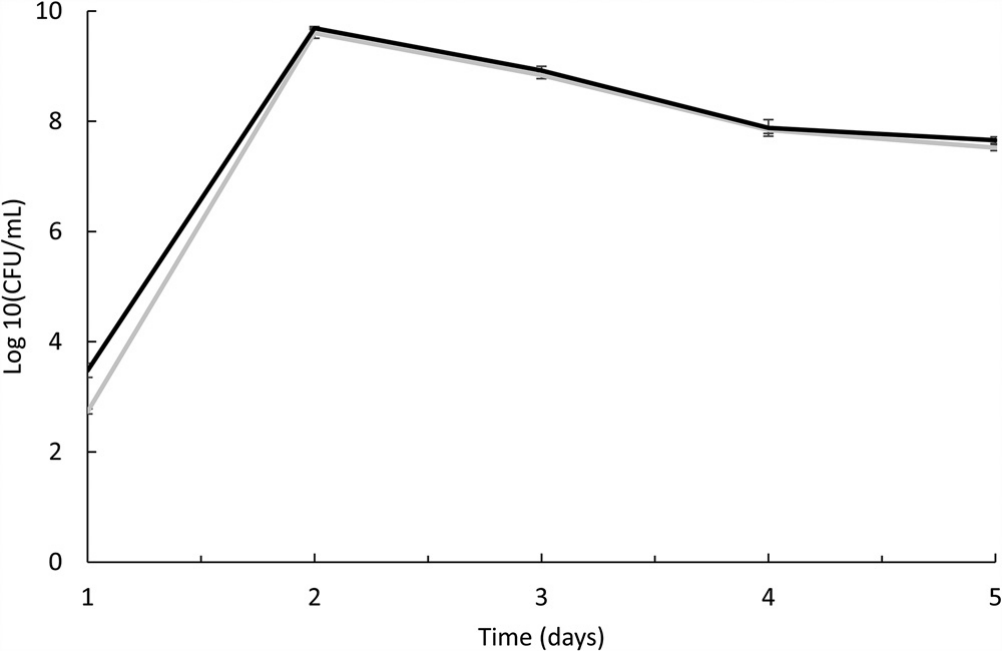
Growth kinetics of Pseudoalteromonas rubra S4059 wild type and prodigiosin-deficient mutant Δ*pigC* when grown in marine minimal medium containing mannose for 4 days. Gray line: wild-type S4059. Black line: Δ*pigC*. Each experiment is repeated in bio-triplicates and error bars represent standard deviation.

Abolishing prodigiosin production did not alter growth of *P. rubra* S4059, as the wild type and the Δ*pigC* mutant grew with similar growth rate and maximum cell density in marine minimal medium (MMM) with mannose (Fig. 3). The biofilm formation, as determined by crystal violet staining, was also similar in the wild-type S4059 and the mutant (Fig. S1). Furthermore, deletion of a *pigC* gene did not change fitness of the bacteria, as the mutant and wild type coexisted in equal levels in mixed cultures (Fig. S2A and B).

### Antibacterial activity of wild-type S4059 and prodigiosin-deficient mutant Δ*pigC*.

**FIG 4:**
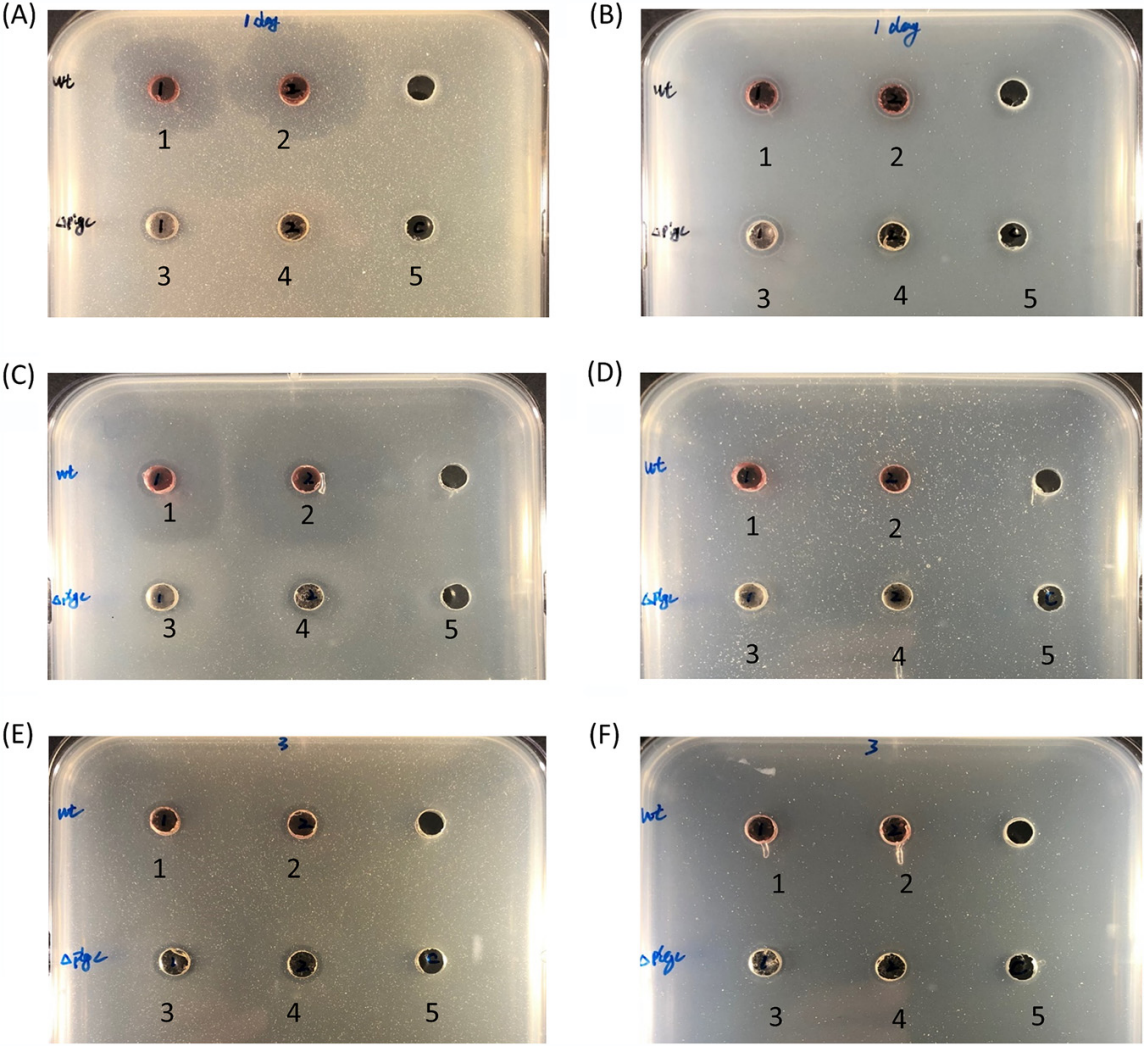
Screening Pseudoalteromonas rubra S4059 wild type (1 and 2) and prodigiosin-deficient mutant Δ*pigC* (3 and 4) for antibacterial activity against Staphylococcus aureus 8325 (A, C, E) and Vibrio anguillarum 90-11-287 (B, D, F). Wild-type S4059 and the Δ*pigC* were cultured in marine minimal medium for 24 h, 48 h, or 72 h. A and B, 24 h; C and D, 48 h; E and F, 72 h. 5: medium extractions, negative control. Twenty-milliliter cultures were taken and extracted from each time point for bioassay.

Prodigiosin has pronounced antibacterial activity, and to investigate whether S4059 could produce other antibacterial compounds, ethyl acetate extracts and culture supernatants of wild-type S4059 and Δ*pigC* (sampled after 24 h, 48 h, and 72 h) were tested for antibacterial activity. Extractions from the wild type were antibacterial (Fig. 4); however, extractions from the prodigiosin-deficient mutant Δ*pigC* retained some antibacterial activity (Fig. 4).

### A blue pigment, di-pyrrolyl-dipyrromethene prodigiosin, was produced from the prodigiosin-deficient mutant Δ*pigC*.

**FIG 5:**
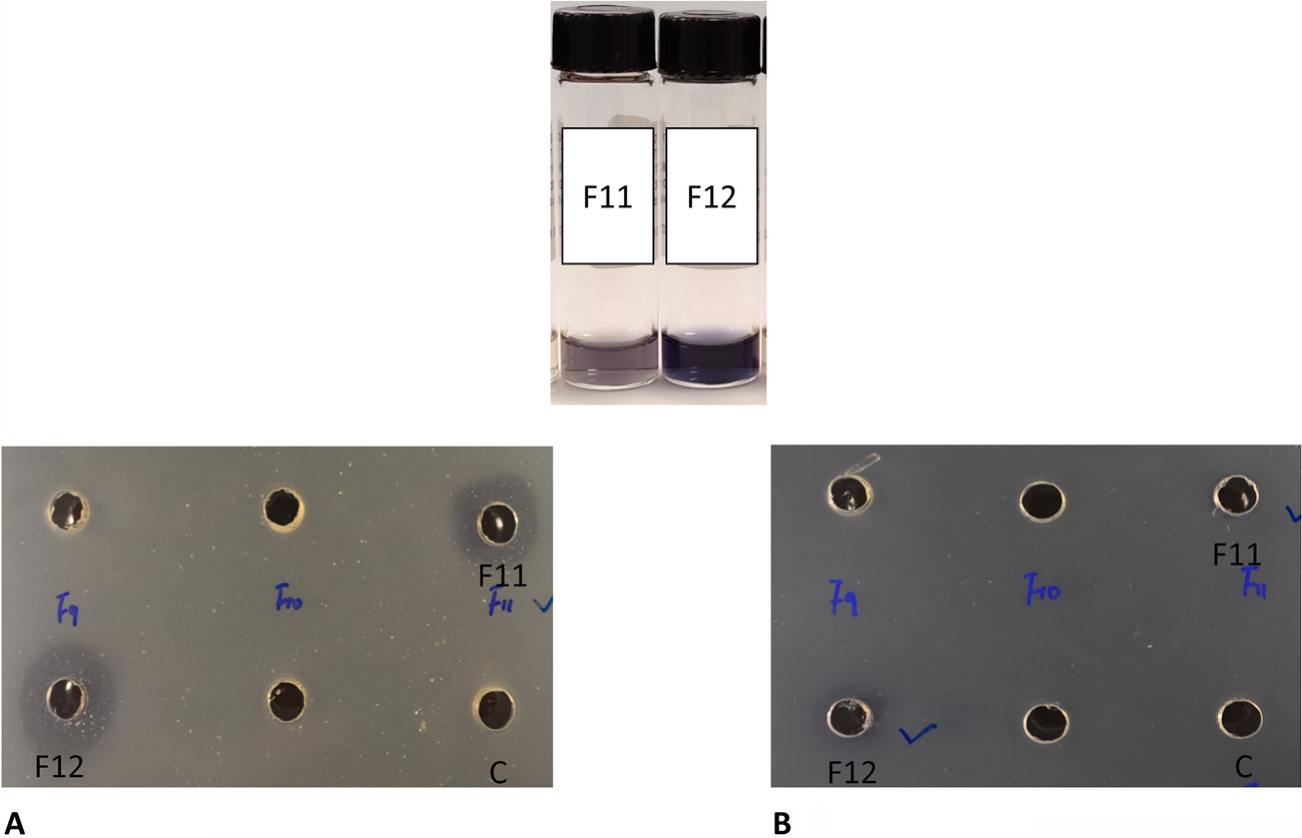
A total of 29 fractions were obtained from the ethyl acetate extract of 500 ml of the prodigiosin-deficient mutant Δ*pigC* and used for disk diffusion assays. Only two fractions, fraction 11 (F11) and fraction 12 (F12), which are two blue fractions, showed antibacterial activity against both Staphylococcus aureus 8325 (A) and Vibrio anguillarum 90-11-287 (B). C: extracts from marine minimal medium containing mannose as a negative control.

Bioassay-guided fractionation of ethyl acetate extracts of the prodigiosin-deficient mutant Δ*pigC* (grown in MMM with mannose) resulted in 29 fractions that were tested in a disk diffusion assay. Two fractions, fractions 11 and 12, had antibacterial activity and were also characterized by having a clear blue color (Fig. 5). The blue fractions 11 and 12 were analyzed by ultra-high-performance liquid chromatography coupled to diode-array detection and high-resolution mass spectrometry (UHPLC-DAD-HRMS), resulting in similar profiles, although fraction 12 was more concentrated. Both fractions contained 4-methoxy-2,2′-bipyrrole-5-carbaldehyde (*m/z* = 191.0822, C_10_H_10_N_2_O_2_, mass accuracy = 3.66 ppm) as the major component. However, MBC has an absorption maximum around 360 nm, which is expected to result in a yellow color (Fig. S3). Further inspection of the obtained data from fraction 12 led to identification of two peaks corresponding to (i) the previously reported blue tetrapyrrole pigment, di-pyrrolyl-dipyrromethene prodigiosin (Dip-PDG), formed via condensation of two MBC molecules (6, 16, 19), as well as (ii) a tentative nonmethylated analogue (Dip-PDG: *m/z* = 335.1509, C_19_H_18_N_4_O_2_, mass accuracy = 1.79 ppm; de-methyl-Dip-PDG: *m/z* = 307.1199, C_17_H_14_N_4_O_2_, mass accuracy = 2.93 ppm) (Fig. S2). Dip-PDG was previously isolated from a 2-methyl-3-*n*-amyl-pyrrole (MAP) pathway-blocked mutant of the bacterium Serratia marcescens 9-3-3 and later reported from the wild type of marine bacterium Hahella chejuensis KCTC 2396 (6, 16). Although we did not isolate these two compounds in pure form, our results indicate that Dip-PDG and the tentative dimethyl-Dip-PDG are likely responsible for the color of the two blue fractions. Neither Dip-PDG nor the proposed analogue was produced by the wild type, and only in extremely small amounts in the mutant, thus not allowing for purification and further studies of the compounds.

### The filtered supernatants from wild-type S4059 and prodigiosin-deficient mutant Δ*pigC* have antibacterial activity.

**FIG 6:**
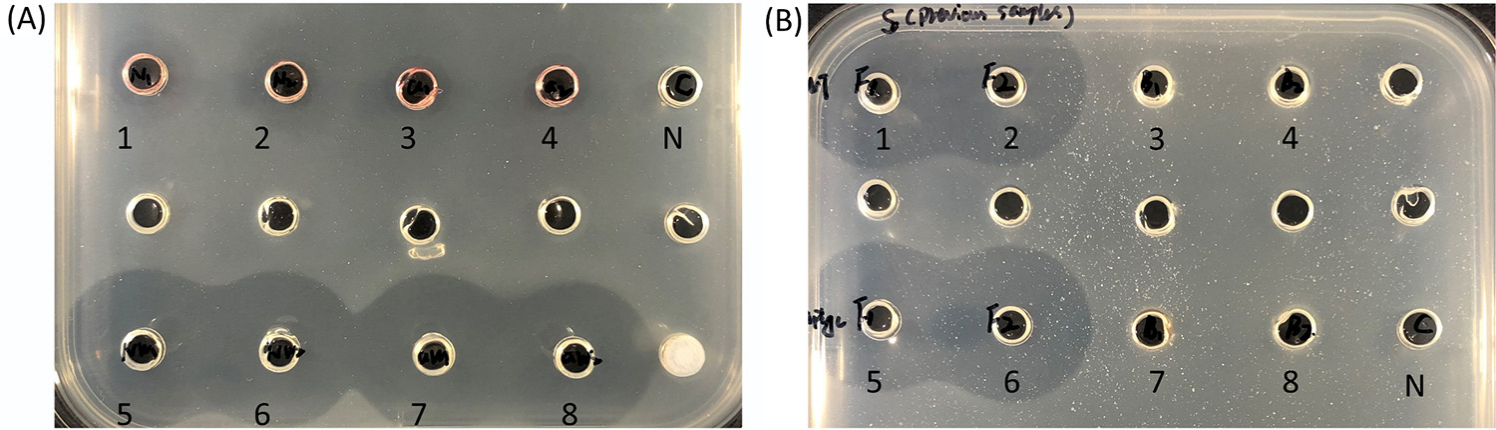
(A) Pseudoalteromonas rubra wild-type S4059 was cultured in marine minimal medium containing mannose. A total of 5 ml of the cultures was extracted with the equal volume of ethyl acetate with or without 1% formic acid, and 20 μl of each extract was tested for antibacterial activity against Staphylococcus aureus 8325. A total of 20 μl liquid from water phase after the extraction also was tested against the two bacteria. 1, 2: wild-type culture extracts using ethyl acetate with 1% formic acid. 3, 4: wild-type culture extracts using ethyl acetate; 5, 6: liquid from water phase after extraction using ethyl acetate. 7, 8: liquid from water phase after extraction using ethyl acetate with 1% formic acid. (B) Pseudoalteromonas rubra wild-type S4059 and prodigiosin-deficient mutant Δ*pigC* were cultured in marine minimal medium containing mannose. One-milliliter culture supernatants were filtered and then treated without or with heating at 95°C for 10 min. Forty-microliter supernatants were tested against Staphylococcus aureus 8325. 1, 2: filtered wild-type supernatants. 3, 4: filtered wild-type supernatants after heating. 5, 6: filtered Δ*pigC* supernatants. 7, 8: filtered Δ*pigC* supernatants after heating.

Not all compounds are extracted by ethyl acetate, and we also tested culture supernatant as well as the water phase following organic solvent extraction for antibacterial activity. The antibacterial activity was similar in extractions with and without formic acid (Fig. 6); however, prodigiosin was more efficiently extracted using acidified ethyl acetate (Fig. S4). Samples of the water phase of the extractions and the culture supernatants were antibacterial (Fig. 6), and the antibacterial activity passed through a 100-kDa filter but was retained on a 30-kDa filter (Fig. S5A and B). The activity was lost upon heat treatment of the supernatant (Fig. 6) but not by treatment with proteinase K or pepsin (Fig. S4). SDS-PAGE analysis of the molecular mass of the bioactive extract in the culture supernatants revealed multiple bands of 15 to 100 kDa (Fig. S5C). According to previous proteomic data on *P. rubra* S4059 (data are available via ProteomeXchange with identifier PXD023249), four extracellular proteases with high relative abundance were found in the samples of wild-type S4059 culture supernatants, including two peptidases (A0A5S3UZL3 and A0A5S3V0P4), one putative alkaline serine protease (A0A5S3V092), and a putative serine protease (A0A5S3UW99) (20). The predicted molecular weights of those proteases were 53 to 64 kDa without signal peptides.

## DISCUSSION

Prodigiosin and its analogs have medicinal potential due to their antibacterial, anticancer, and immunosuppressive properties. Here, we identified a gene involved in the biosynthesis of prodigiosin and also, through sequence homology, other genes in the BGC likely related to the biosynthetic pathway in *P. rubra* S4059. We also, for the first time, reported the production of a stable blue antibacterial pigment, Dip-PGD, in the prodigiosin-deficient mutant of Pseudoalteromonas rubra S4059.

Recently, a phylogenetic study on the evolution of the prodigiosin BGC in several bacteria, including the genera *Serratia* and *Pseudoalteromonas*, was conducted by Ravindran et al. (21), and the authors suggested that these bacteria contained similar proteins in the prodigiosin BGCs and that they had originated from a common ancestor. In our study, *in silico* comparison between the suggested prodigiosin BGC in S4059 and *Serratia* sp. ATCC 39006 found a high homology of the *pig* operon in the genomes of the two strains, similarly suggesting a common ancestor of the prodigiosin BGCs in the two strains.

The biosynthesis of prodigiosin has been studied in S. marcescens, and the production involves a coupling step of two precursors: MBC and MAP. The prodigiosin-synthesizing transferase PigC is the condensing enzyme in *Serratia* sp. ATCC 39006 (22). Both of the two precursor molecules can be detected in the ATCC 39006 *pigC* mutant, indicating that the deletion of *pigC* does not disrupt the biosynthesis of the two precursors and only disrupts the formation of prodigiosin (22). In our study, the *pigC* gene was selected as a target for identifying the prodigiosin BGC in S4059 and a *pigC* in-frame deletion mutant was generated in *P. rubra* S4059. Similar to those in the *Serratia* sp. ATCC 39006 *pigC* mutant, the prodigiosin production was abolished and a large amount of accumulated MBC was detected in the culture extracts of S4059 Δ*pigC* mutant. However, MAP was not detected in the S4059 Δ*pigC* mutant cultures. This could be due to the volatility of MAP, as reported from another marine bacterium, Hahella chejuensis KCTC 2396 (6). Furthermore, two fractions with antibacterial activity and blue color were obtained by fractionation of the culture extracts of the S4059 Δ*pigC* mutant. Chemical analysis showed that MBC was the dominant compound in the two fractions and that both fractions contained small amounts of Dip-PDG as well as a tentative nonmethylated analogue. Both MBC and Dip-PDG contain methoxy groups that contribute to the bioactivity of prodigiosin, suggesting that MBC and Dip-PDG are indeed the bioactive compounds (6, 23); however, further work is needed to determine the exact nature of their antibacterial activity.

Antibacterial activity is caused not only by “small molecules” but can also be caused by proteins, including proteolytic enzymes (24). For instance, Pseudoalteromonas piscicida can kill competing bacteria by secretion of proteolytic enzymes, such as a trypsin-like serine protease (25), and similar mechanisms of antibacterial activity have also been found in other *Pseudoalteromonas* species (26). In this study, the filtered aqueous supernatants from both the wild type and the Δ*pigC* mutant exhibited a high antibacterial activity that was lost after heat treatment, suggesting that unextractable proteins/peptides or compounds with the currently used methods may be responsible for the activities. Three putative RiPP-like proteins were predicted in the genome of S4059 by antiSMASH 6.0 (27), and they all contain a DUF692 domain which is found in bacteriocin BGCs (28, 29), suggesting that bacteriocins could be responsible for the antibacterial activity in the supernatants. However, the antibacterial activity of the supernatants was not abolished by treatment with proteinase K or pepsin (Fig. S4). Not all bacteriocins are inactivated by treatment with proteinase K and pepsin as reported for several lactic acid bacteria (30). Previous proteomic analysis on *P. rubra* S4059 (20) (data are available via ProteomeXchange with identifier PXD023249) indicated that the strain produced several proteases that may have the same antibacterial function as that described in P. piscicida (25). Further studies with the molecular weight cutoff and protein analyses found that the antimicrobial activities may also be due to extracellular proteases/peptidases, which were often secreted by marine bacteria and reported with potent antibacterial activity (31–33).

In conclusion, we succeeded in identifying the prodigiosin BGC in Pseudoalteromonas rubra S4059, and a stable blue pigment was for the first time reported in the *pigC* mutant of S4059. These findings provide essential information for further exploring the industrial potential of prodigiosin and its analogs.

## MATERIALS AND METHODS

### Bacterial strains, plasmids, and cultural conditions.

The bacterial strains and plasmids used in this study are listed in Table S2. *P. rubra* S4059 was isolated during the Galathea 3 expedition (34) and cultured on marine agar (MA; BD Difco 2216) or in marine broth (MB; BD Difco 2216) at 25°C. Liquid cultures were incubated at 25°C with shaking at 200 rpm. *P. rubra* S4059 wild type and prodigiosin-deficient mutant Δ*pigC* were cultured in marine minimal medium (MMM) (35) supplemented with 0.2% (wt/vol) mannose at 25°C and 200 rpm for chemical extraction. The composition of the medium for antibacterial activity assay against Gram-negative bacterium Vibrio anguillarum 90-11-287 and Gram-positive bacterium Staphylococcus aureus 8325 was 1.2 g agar (A0949, ITW reagents), 3.6 g instant ocean salts (Aquarium systems), and 0.4 g casamino acid (BD, 223050) in 120 ml distilled H_2_O. The medium was supplemented with 1.2 g peptone (BD, 211677) in assay with S. aureus as a target organism.

All Escherichia coli strains were cultured in Luria Bertani broth (BD Difco. 244520) at 37°C, 200 rpm. E. coli GB *dir-pir*116 was used for constructing suicide plasmid (36). E. coli WM 3064 is a *dapA* mutant requiring exogenously supplied diaminopimelic acid (DAP; Sigma, D1377) with a final concentration of 0.3 μM for growth (37) and was used as a donor strain for intergeneric conjugation. Plasmid pDM4 (38) was used as the backbone vector to construct suicide plasmids. E. coli strains harboring pDM4 or its derivatives were grown in LB broth with 10 μg/ml chloramphenicol or on LB agar with 15 μg/ml chloramphenicol.

### DNA manipulation.

Genomic DNA of *P. rubra* S4059 was extracted using genomic DNA buffer set (Qiagen, 19060) following the supplier’s instructions. All purified DNA fragments were amplified using PrimeSTAR max premix (TaKaRa, catalog number R045A). Blue TEMPase hot start master mix K (Amplicon, catalog number 733-2584) was used for colony PCR and homologous recombination event checking by PCR. All primers (Table S3) and plasmids were designed in A Plasmid Editor (ApE). The specificity of primers was checked by BLAST against the *P. rubra* S4059 genome. All primers were synthesized by Integrated DNA Technologies (Leuven, Belgium).

### *In silico* analysis of prodigiosin biosynthetic gene cluster.

The genome of *P. rubra* S4059 is available at the National Center for Biotechnology Information (NCBI) under the accession number CP045429. The genome was annotated by Prokka (17). Annotated homologous genes (*pigA* to *pigM*) of prodigiosin biosynthetic gene cluster (BGC) were identified using CLC Main Workbench 8.0.1 (CLC bio, Aarhus, Denmark) and antiSMASH 6.0 bacterial version (27). Homologs of the Pig proteins were compared on MaGe microscope platform (39).

### In-frame deletion of *pigC* gene in *P. rubra* S4059.

The gene knockout strategy is followed from Wang et al. (20). Briefly, approximately 1-kb upstream and downstream regions flanking *pigC* gene were amplified and fused by overlap extension PCR, and the recombining segment was inserted into pDM4 vector to construct the suicide plasmid by direct cloning method (36, 38). The suicide plasmid was transferred from E. coli WM 3064 to *P. rubra* S4059 by intergeneric conjugation. With two-step homologous recombination and counterselection, the mutant was confirmed by diagnostic PCR and DNA fragments sequencing.

### Growth kinetics of *P. rubra* S4059 wild type and prodigiosin-deficient Δ*pigC* mutant and coculture of the two strains.

Precultures of S4059 wild type and Δ*pigC* were incubated in MB overnight at 25°C, 200 rpm. The cultures were diluted to a starting cell density of 10^3^ CFU/ml in 20 ml MMM containing 0.2% mannose. Samples were taken every day to estimate cell density by colony counting on MA plate. For coculture experiment, the precultures of S4059 wild type and Δ*pigC* were diluted to a starting cell density of approximately 10^3^ CFU/ml in the final volume of 10 ml MMM or MB in the same 50 ml Falcon tube. Cell densities were followed by serial dilution and plating on MA.

### Biofilm formations of *P. rubra* S4059 wild type and prodigiosin-deficient Δ*pigC* mutant.

The protocol was modified from O’Toole and Kolter crystal violet assay (40). Briefly, *P*. *rubra* S4059 and Δ*pigC* mutant strain were cultured with MB in 50 ml Falcon tubes at 25°C, 200 rpm overnight. The overnight cultures were diluted to an optical density at 600 nm (OD_600_) of 0.01 using MMM containing mannose in a final volume of 500 μl. Biofilm formation was evaluated in 96-well polystyrene plates (Thermo scientific, 163320), and 100 μl liquid of the diluted cultures was added into each well in bio-triplicates. The plates were incubated at 25°C for 24, 48, or 72 h in a humidity chamber. The biofilm was visualized by crystal violet staining and dissolved in 95% ethanol to measure the absorbance at 590 nm. Fresh medium was used as a negative control.

### Extraction of metabolites for chemical analysis.

The protocol was modified from Wang et al. (20). Briefly, *P. rubra* S4059 and the prodigiosin-deficient mutant Δ*pigC* were cultured in 100 ml MMM containing 0.2% mannose at 25°C, 200 rpm and samples were taken after 1, 2, and 3, days. A total of 20 ml of the culture was extracted with an equal volume of high-performance liquid chromatography (HPLC)-grade ethyl acetate (EtOAc) with or without 1% formic acid in 50-ml Falcon tubes. The organic phase was transferred to a new Falcon tube and evaporated under nitrogen. The dried extracts were redissolved in 200 μl methanol, and extractions were used for antibacterial activity assay and chemical analysis by UHPLC-DAD-HRMS.

### Antibacterial activity assay.

The protocol was modified from Gram et al. (34). Briefly, the extractions of metabolites were tested for antibacterial activity against Vibrio anguillarum 90-11-287 and Staphylococcus aureus 8325. The composition of agar medium was described above, and 6-mm wells were punched in the agar plates with a least 2-cm distances between the wells. A total of 20 μl of extractions or 40 μl of filtered supernatants was pipetted into the well of the plates for bioassay. To verify whether the antibacterial activity of the supernatants was due to unextracted compounds or proteins/peptides, the filtered supernatants were also treated with heating at 95°C, 10 min, and proteinase K (P6556, Sigma) or pepsin (P6887, Sigma). The plates were incubated at 25°C for 24 to 48 h. All experiments were done in biological duplicate.

### Fractionation of extract from prodigiosin-deficient *P. rubra* mutant.

The extract used for fractionation was made using a modified protocol similar to the one used for chemical analysis. Five 100-ml cultures were combined and extracted using an equal amount of EtOAc. The aqueous and organic phases were separated, and the organic phase was evaporated to dryness, yielding 30 mg of crude extract. The extract was redissolved in methanol and fractionated in three 10-mg portions on a Kinetex Core-Shell C_18_ column (250 mm by 10 mm, 5 μm, Phenomenex), coupled to an Agilent Infinity II 1260 preparative HPLC system. The mobile phase was made up of HPLC-grade water (solvent A) and acetonitrile (solvent B), both buffered with 20 mM formic acid (FA). Elution was achieved using a flow rate of 5 ml/min and linear gradient starting with 10% B increasing to 100% in 30 min, collecting time slices of 1 min each.

### Chemical analysis.

Chemical analysis was performed using a maXis 3G orthogonal acceleration quadrupole time-of-flight mass spectrometer (QTOF-MS) (Bruker Daltonics) connected to an Ultimate 3000 UHPLC system (Dionex). Separation was achieved using a Kinetex 1.7-μm C_18_, 100 mm by 2.1 mm column (Phenomenex). The column temperature was maintained at 40°C throughout the analysis, and a linear gradient using LC-MS-grade water and acetonitrile both buffered with 20 mM LC-MS-grade formic acid, starting from 10% (vol/vol) acetonitrile and increased to 100% in 10 min, maintaining this rate for 3 min before returning to the starting conditions in 0.1 min and staying there for 2.4 min before the following run. A flow rate of 0.4 ml · min^−1^ was used. Mass spectrometric detection was performed in ESI^+^ with a data acquisition frequency of 10 scans per second in the *m/z* range 75 to 1,250. Mass calibration was done using Bruker Daltonics high-precision calibration algorithm by automatically infusing the internal standard sodium formate before each run. UV-visible (UV-Vis) absorption spectra were collected at wavelengths from 190 to 800 nm. Data processing and analysis were performed using DataAnalysis 4.0.

### Concentration of proteins in the culture supernatant.

*P. rubra* S4059 wild type and Δ*pigC* strains were grown in MMM containing 0.2% mannose for 2 days at 25°C with shaking at 200 rpm. The culture supernatants were collected by filtering through 0.2-μm filters, and the concentration of the antimicrobial components in the culture supernatants was performed according to the user guide of Thermo Scientific Pierce protein concentrators. The culture supernatant of 20-fold concentration was achieved by passing the supernatants through a 100-kDa molecular weight cutoff (100-K MWCO) membrane (Thermo Scientific, catalog number 88523). The retention was collected, and part of the permeate was then pumped through a 30-K MWCO membrane (Thermo Scientific, catalog number 88521). The permeates from 100-K and 30-K MWCO membranes were also collected for further tests. The antimicrobial activity of each portion was performed as described above.

### Detection of proteins by SDS-PAGE and staining.

All fractions from the protein concentration step were collected, and 30 μl of each was prepared by mixing with 4× Laemmli sample buffer with β-mercaptoethanol (Bio-Rad, 1610747) followed by boiling at 95°C for 5 min. All samples were loaded to a 4% to 12% Mini-PROTEIN TGX precast gel (Bio-Rad, 4561093), and the precision plus protein dual-color standard (Bio-Rad, catalog number 1610374) was used for molecular weight estimation. Proteins were separated at 60 V for 50 min followed by a migration at 150 V for 50 min before staining in InstantBlue Coomassie protein stain (Abcam, ISB1L, ab119211) for half an hour.
