# Supplementary material for: Identification and Verification of the Prodigiosin Biosynthetic Gene Cluster (BGC) in Pseudoalteromonas rubra S4059
Source: Microbiol Spectr. 2021 Sep 29;9(2):e01171-21. doi: 10.1128/Spectrum.01171-21 (PMC8557933; doi:10.1128/Spectrum.01171-21)
Supplement: SUPPLEMENTAL FILE 1 — Supplemental material. Download SPECTRUM01171-21_Supp_1_seq10.pdf, PDF file, 0.3 MB [file spectrum01171-21_supp_1_seq10.pdf]

Identification and verification of the prodigiosin biosynthetic gene cluster (BGC) in *Pseudoalteromonas rubra* S4059

Xiyan Wang, Thomas Isbrandt, Emil Ørsted Christensen, Jette Melchiorsen, Thomas Ostenfeld Larsen, Sheng-Da Zhang and Lone Gram *

Department of Bioengineering, Technical University of Denmark, DK-2800 Kgs. Lyngby, Denmark; xwan@dtu.dk (X.W.); tispe@bio.dtu.dk (T.I.); emiloersted@live.dk (E. Ø. C); jeme@bio.dtu.dk (J.M); tol@bio.dtu.dk (T.O.L); shez@dtu.dk (S.Z.).

*** Corresponding author:**

Lone Gram

Department of Bioengineering, Technical University of Denmark, DK-2800 Kgs. Lyngby, Denmark

gram@bio.dtu.dk (L.G.)

Running title: Identification of prodigiosin BGC in *P. rubra* S4059.

Keywords: Biosynthetic gene cluster, dipyrrolyldipyrromethene prodigiosin, prodigiosin, *Pseudoalteromonas rubra*.

SUPPLEMENTARY MATERIALS TABLE AND FIGURE LEGENDS

Table S1. The percent identities of gene products amongst *Serratia* sp. ATCC 39006, *Pseudoalteromonas* sp. R3 and *Pseudoalteromonas rubra* S4059. Putative functions were assigned based on the results of BLASTp searches.

Table S2. The bacterial strains and plasmids used in this study.

Table S3. Primers used in this study.

Figure S1. Biofilm formation of *Pseudoalteromonas rubra* S4059 wild type and prodigiosin deficient mutant Δ*pigC* in marine minimal medium (MMM) or marine both (MB) for 24 h (A and B), 48 h (C and D), and 72 h (E and F) as determined by the O'Toole & Kolter crystal violet assay. Each experiment is repeated in bio-triplicates and error bars are standard error. Wt: wild type S4059; pigC: prodigiosin deficient mutant Δ*pigC*.

Figure S2. Co-culture of *Pseudoalteromonas rubra* S4059 wild type and prodigiosin deficient mutant Δ*pigC* in marine minimal medium (MMM, A) or marine both (MB, B). Grey lane: wild type S4059; black lane: Δ*pigC*. Each experiment is repeated in bio-triplicates and error bars are standard deviation.

Figure S3. (A) UV-Vis absorption spectrum for MBC (m/z 191.0822) measured during the chromatographic analysis. (B) Base peak chromatogram (BPC) of fraction 12, indicating proposed identity of tentatively identified metabolites.(C) the chemical structure of Prodigiosin, (4-methoxy-2,2'- bipyyrrole-5-carbaldehyde (MBC) and Dipyrrolyldipyrromethene prodigiosin (Dip-PDG).

Figure S4. *Pseudoalteromonas rubra* S4059 was cultured in marine minimal medium containing mannose and the cultures were extracted using ethyl acetate with (A) or without 1% formic acid (B). The filtered supernatants (FSNs) of wild type *P. rubra* S4059 (1, 2) and *ΔpigC* mutant (3, 4) were treated with proteinase K (Pk) or pepsin (Pn) and still retain anti- *Staphylococcus aureus* 8325 activities (C).

Figure S5. The culture supernatants of *Pseudoalteromonas rubra* S4059 wild type and ΔpigC strains were concentrated by 100 and 30 kDa WMCO filters. The retentions and permeates were tested for the antimicrobial activity against *Vibrio anguillarum* strain 90-11-286 (A) and *Staphylococcus aureus* 8325 (B). The bioactive retentions were then analyzed by SDS-PAGE gel electrophoresis (C). Proteins were detected with InstantBlue staining.

Table S1. The percent identities of gene products amongst *Serratia* sp. ATCC 39006, *Pseudoalteromonas* sp. R3 and *Pseudoalteromonas rubra* S4059. Putative functions were assigned based on the results of BLASTp searches.

Gene	Locus_tag			*Pseudoalteromonas. rubra* S4059 proteins vs *Serratia* sp. ATCC 39006 proteins (Homologs (identity %)	*Pseudoalteromonas. rubra* S4059 proteins vs *Pseudoalteromonas.* sp. R3 proteins (Homologs (identity %)	Predicted protein function
	Pseudoalteromonas rurba S4059	*Serratia* sp. ATCC 39006	*Pseudoalteromonas* sp. R3			
PigA	CWC22_013905	WP_021014639	WP_054014157	69.19	94.01	L-prolyl-PCP dehydrogenase
PigB	CWC22_013910	WP_021014640	WP_054014158	62.29	87.69	Oxidoreductase
PigC	CWC22_013915	WP_021014641	WP_054014159	73.3	96.26	Terminal Condensation enzyme
PigD	CWC22_013920	WP_021014642	WP_054014160	74.46	95.06	Thiamine diphosphate dependent-3-acetyloctanal synthase
PigE	CWC22_013925	CAH55633	WP_054014161	79.46	96.71	Aminotransferase
PigF	CWC22_013930	WP_021014644	WP_054014162	78.99	97.64	S-adenosyl-L-methionine-dependent methyltransferase
PigG	CWC22_013935	WP_021014645	WP_054014163	74.36	93.9	putative acyl carrier protein
PigH	CWC22_013940	WP_021014646	WP_054014164	71.82	94.29	HBM synthase/aminotransferase
PigI	CWC22_013945	WP_021014647	WP_054014165	60.25	85.54	L-prolyl-AMP ligase
PigJ	CWC22_013950	CAH55638	WP_054014166	60.76	81.13	β-ketoacylsynthase

PigK	CWC22_013955	WP_021014649	WP_054014167	66.67	93.27	Hypothetical protein (RedY)
PigL	CWC22_013960	WP_051391462	WP_054014168	36.9	56.22	Phosphopantetheinyl transferase
PigM	CWC22_013965	CAH55641	WP_054014169	53.54	82.22	Putative NAD(P)H nitroreductase

Table S2. The bacterial strains and plasmids used in this study

Strains/Plasmids	Genotype or relevant characteristics	Reference or source
Escherichia coli		
WM3064	thrB1004 pro thi rpsL hsdS lacZΔM15 RP4-1360 Δ(araBAD)567 ΔdapA1341::[erm pir], 37 ℃. Donner strains in conjugation.	(1)
GB *dir pir*116	An arabinose-inducible *ETγA* operon (full-length *recE*, *recT*, *redγ*, and *recA*), a copy-up *pir*116 gene. Host strain for constructing suicide plasmids with R6K replication origin.	(2)
Pseudoalteromonas rubra		
S4059	Wild type strain, Isolated from seaweed.	(3)
Δ*pigC*	*PigC* gene in-frame deletion mutant of *P. rubra* S4059 Δ *PigC*	This study
Vibrio anguillarum		
90-11-287	Serotype O1 pathogenic to fish larvae	(4)
Staphylococcus aureus		
NCTC 8325		(5)
**Plasmids**		
pDM4	Suicide vector for targeted mutagenesis; *sac*B; *ori*R6K; Cm^R	(6)
pDM4 –del-*pigC*	The left arm and right arm DNA region of the *pigC* gene were PCR amplified from *P. rubra* S4059 genome and cloned in the pDM4 plasmid by direct cloning	This study

Table S3. Primers used in this study.

Primer	Sequence	Description	Expected size (bp)
pigC-L-F	ATCTGTTCGAGCCGGAAA	Left arm of pigC	1000
pigC -L-R	GGATTAGATATACCGTCTCCTTATTAC GCG		
pigC -R-F	AGGAGACGGTATATCTAATCCATCGC GGGC	Right arm of pigC	1017
pigC -R-R	TTTGGACCACGTAGCACC		
Cm^R F	GGCATTTCAGTCAGTTGCTC	Detection of Cm^R gene	525
Cm^R R	CCATCACAAACGGCATGATG		
pigC-pDM4-F	GGTGCTACGTGGTCCAAAGGTCGAC GGTATCGATAAG	Linear pDM4 plasmid (pigC)	7106
pigC-pDM4-R	TTTCCGGCTCGAACAGATTAGATCTT GCATGCGGGT		
pigC -p 1	GGTTAACAGGCCAGCTACTG	Confirmation of pigC mutant	1st: P1, P2 3879[a]; 1230[b]
pigC -p 2	GCCCGCGATGGATTAGA		P3, P4 3878[a] ; 1229[b]
pigC -p 3	CGCGTAATAAGGAGACGGTATA		2nd: P1, P2 3879[a] ; 1230[b]
pigC -p 4	GGTGCCCTGAAAGCTATACC		

[a] the size of WT; [b] the size of mutant.

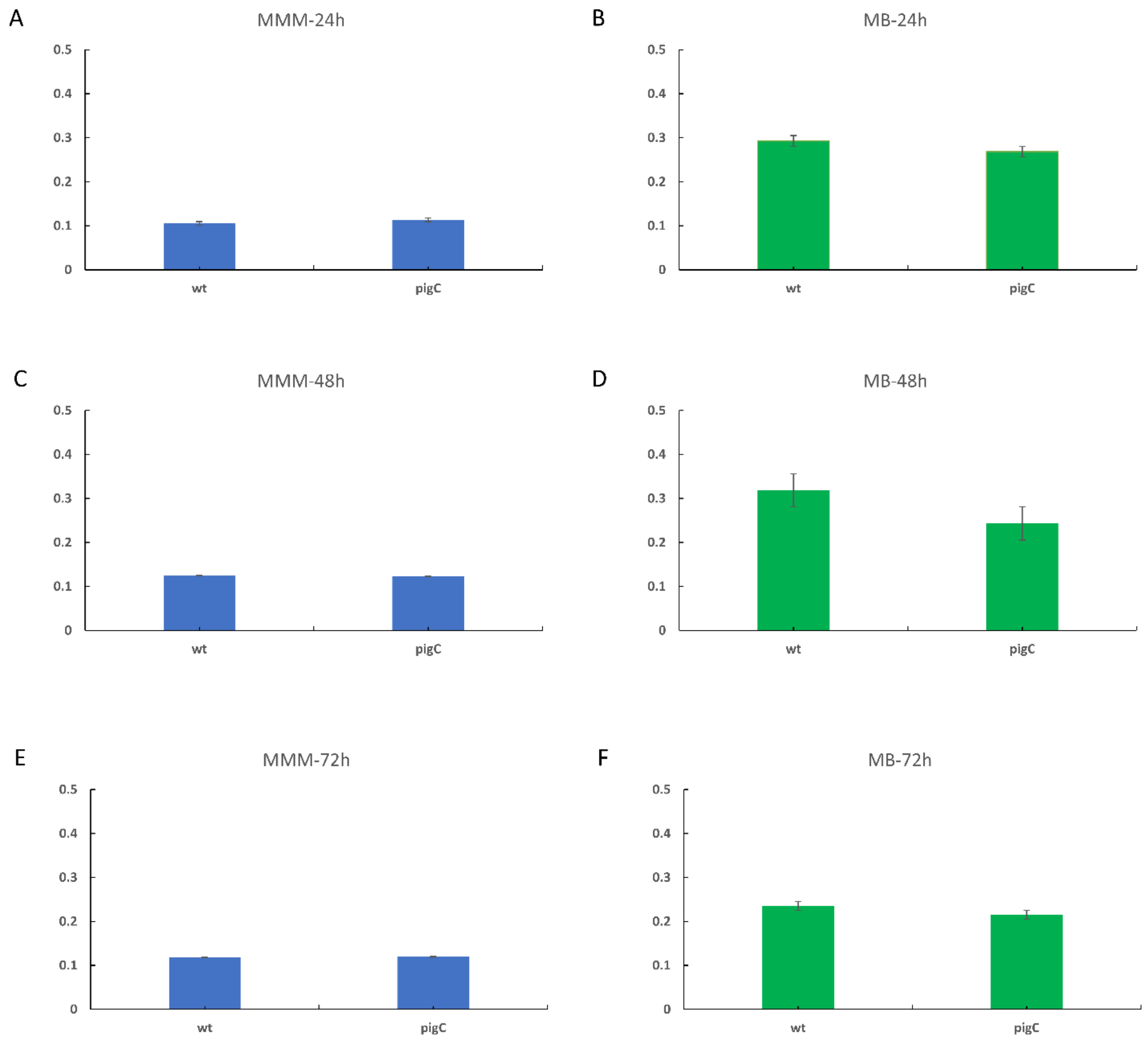

Figure S1. Biofilm formation of *Pseudoalteromonas rubra* S4059 wild type and prodigiosin deficient mutant Δ*pigC* in marine minimal medium (MMM) or marine both (MB) for 24 h (A and B), 48 h (C and D), and 72 h (E and F) as determined by the O'Toole & Kolter crystal violet assay. Each experiment is repeated in bio-triplicates and error bars are standard deviation. Wt: wild type S4059; pigC: prodigiosin deficient mutant Δ*pigC*.

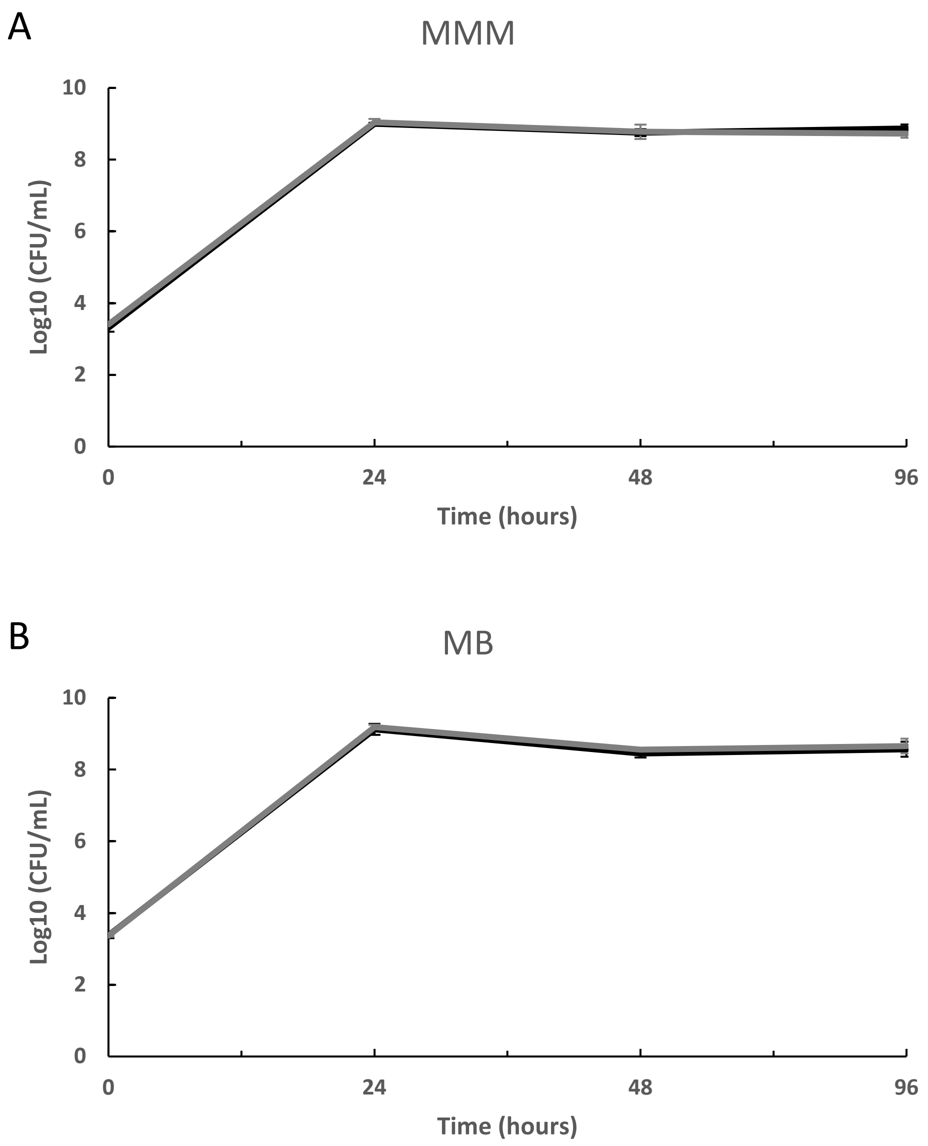

Figure S2. Co-culture of *Pseudoalteromonas rubra* S4059 wild type and prodigiosin deficient mutant Δ*pigC* in marine minimal medium (MMM, A) or marine both (MB, B). Grey lane: wild type S4059; black lane: Δ*pigC*. Each experiment is repeated in bio-triplicates and error bars are standard deviation.

A

B

C

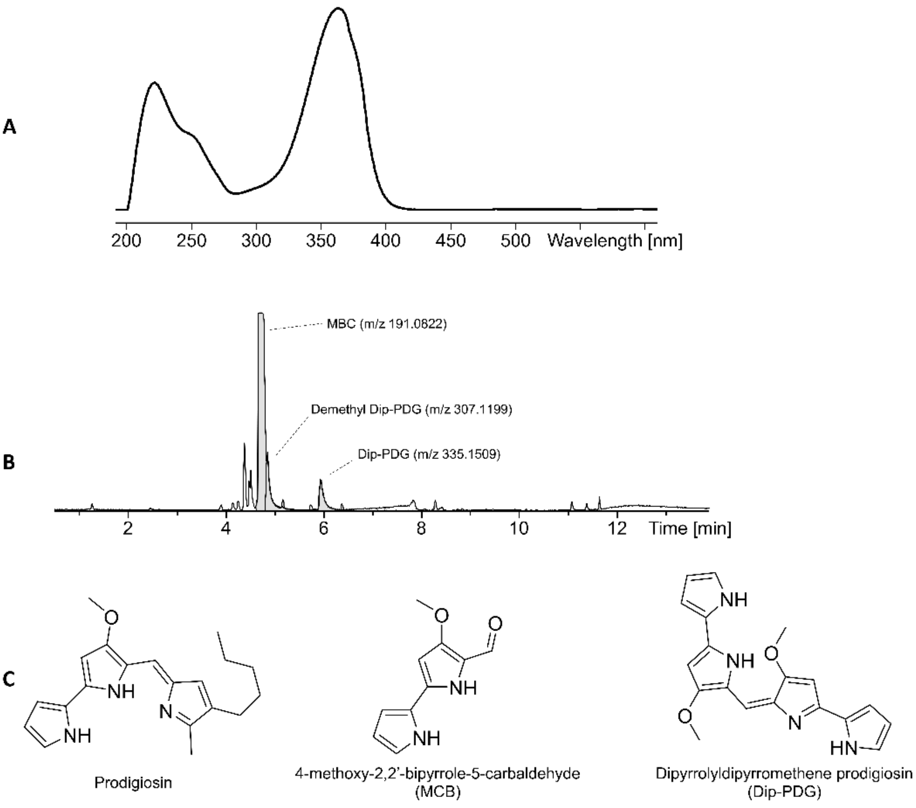

Figure S3. (A) UV-Vis absorption spectrum for MBC (m/z 191.0822) measured during the chromatographic analysis. (B) Base peak chromatogram (BPC) of fraction 12, indicating proposed identity of tentatively identified metabolites.(C) the chemical structure of Prodigiosin, (4-methoxy-2,2'- bipyyrrole-5-carbaldehyde (MBC) and Dipyrrolyldipyrromethene prodigiosin (Dip-PDG).

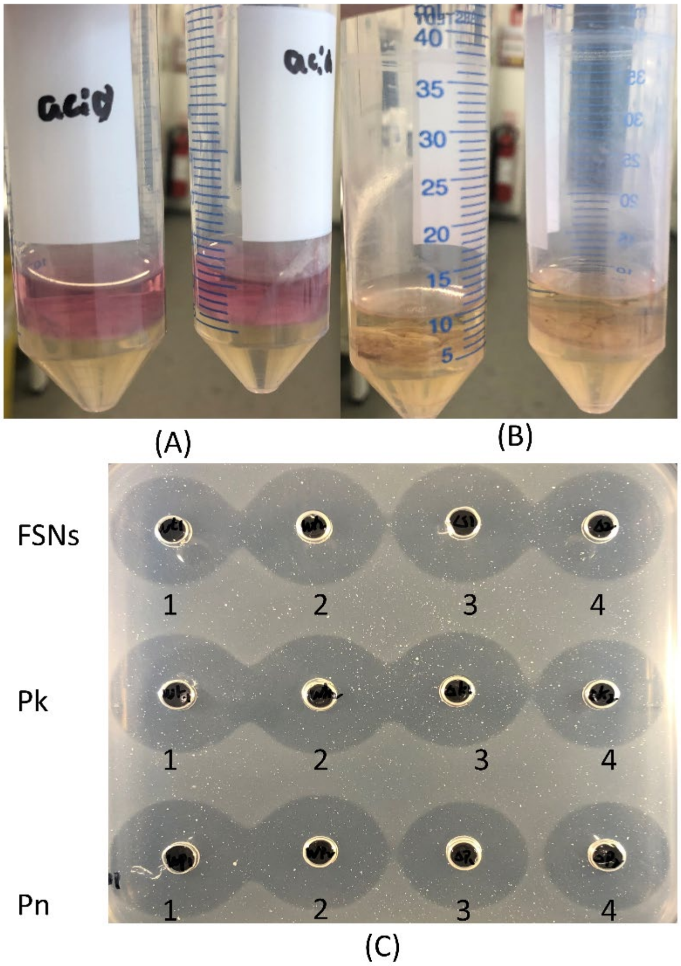

Figure S4. *Pseudoalteromonas rubra* S4059 was cultured in marine minimal medium containing mannose and the cultures were extracted using ethyl acetate with (A) or without 1% formic acid (B). The filtered supernatants (FSNs) of wild type *P. rubra* S4059 (1, 2) and *pigC* mutant (3, 4) were treated with proteinase K (Pk) or pepsin (Pn) and still remain anti- *Staphylococcus aureus* 8325 activities (C).

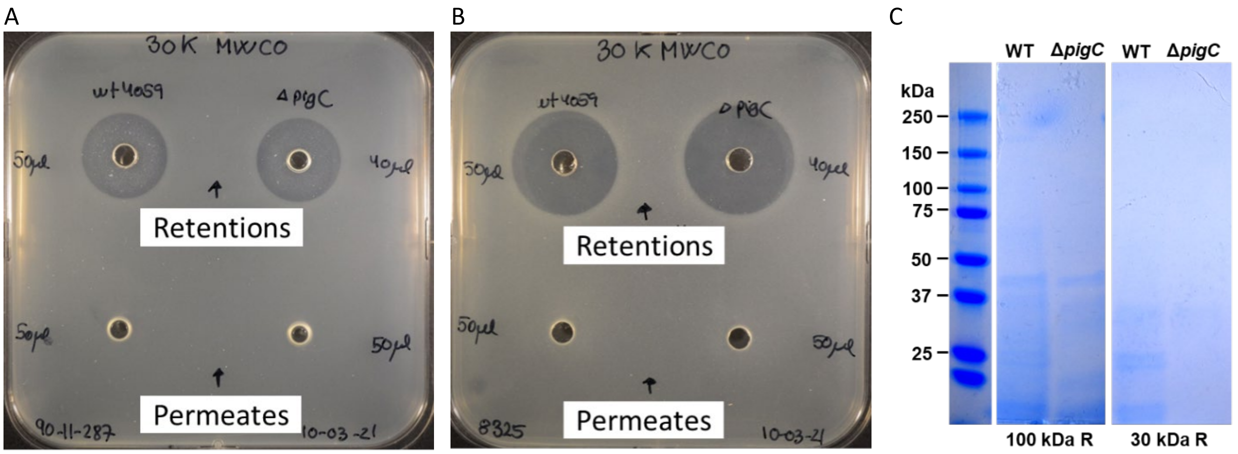

Figure S5. The culture supernatants of *Pseudoalteromonas rubra* S4059 wild type and *ΔpigC* strains were concentrated by 100 and 30 kDa WMCO filters. The retentions and permeates were tested for the antimicrobial activity against *Vibrio anguillarum* strain 90-11-286 (A) and *Staphylococcus aureus* 8325 (B). The bioactive retentions were then analyzed by SDS-PAGE gel electrophoresis (C). Proteins were detected with InstantBlue staining.

References

1. Dehio C, Meyer M. 1997. Maintenance of broad-host-range incompatibility group P and group Q plasmids and transposition of Tn5 in *Bartonella henselae* following conjugal plasmid transfer from *Escherichia coli*. J Bacteriol 179:538–540.

2. Wang H, Li Z, Jia R, Hou Y, Yin J, Bian X, Li A, Müller R, Stewart AF, Fu J, Zhang Y. 2016. RecET direct cloning and Redαβ recombineering of biosynthetic gene clusters, large operons or single genes for heterologous expression. Nat Protoc 11:1175–1190.

3. Gram L, Melchiorsen J, Bruhn JB. 2010. Antibacterial activity of marine culturable bacteria collected from a global sampling of ocean surface waters and surface swabs of marine organisms. Mar Biotechnol 12:439–451.

4. Skov MN, Pedersen K, Larsen JL. 1995. Comparison of pulsed-field gel electrophoresis, ribotyping, and plasmid profiling for typing of *Vibrio anguillarum* Serovar O1. Appl Environ Microbiol 61:1540–1545.

5. Novick R. 1967. Properties of a cryptic high-frequency transducing phage in *Staphylococcus aureus*. Virology 33:155–166.

6. Milton DL, O’Toole R, Hörstedt P, Wolf-Watz H. 1996. Flagellin A is essential for the virulence of *Vibrio anguillarum*. J Bacteriol 178:1310–1319.
